# Supplementary material for: Precision medicine and actionable alterations in lung cancer: A single institution experience
Source: PLoS One. 2020 Feb 11;15(2):e0228188. doi: 10.1371/journal.pone.0228188 (PMC7012442; doi:10.1371/journal.pone.0228188)
Supplement: S1 Table — (DOCX) [file pone.0228188.s001.docx]

**S1 Table.** NGS platforms of testing.

| Test Name | Facility |
| --- | --- |
| FoundationOne | Foundation Medicine, Cambridge, MA |
| Onco48 | City of Hope, Duarte, CA |
| Response DX: Lung | Cancer Genetics, Los Angeles, CA |
| LabCorp | LabCorp, Burlington, NC |
| OncoComplete | City of Hope, Duarte, CA |
| Caris | Caris Life Sciences, Dallas, TX |
| MD Anderson | MD Anderson, Houston, Texas |
| Mayo Clinic | Mayo Clinic, Rochester, MN |
| Hopeseq Lung | City Of Hope, Duarte, CA |
| Guardant 360 | Guardant Health, Redwood City, CA |
| bioT3 | bioTheranostics, San Diego, CA |
