# Supplementary material for: Precision medicine and actionable alterations in lung cancer: A single institution experience
Source: PLoS One. 2020 Feb 11;15(2):e0228188. doi: 10.1371/journal.pone.0228188 (PMC7012442; doi:10.1371/journal.pone.0228188)
Supplement: S2 Table — (DOCX) [file pone.0228188.s002.docx]

**S2 Table.** Actionable genomic alteration rates in TCGA and GENIE.

| Actionable Alterations | TCGA  (n=507) | | | GENIE  (n=6529) | | |
| --- | --- | --- | --- | --- | --- | --- |
|  | Alteration | Tested | Alteration Rate | Alteration | Tested | Alteration Rate |
| EGFR L858/exon 19 Deletion | 46 | 507 | 9.1% | 1117 | 6529 | 17.1% |
| ROS1 Rearrangement | 7 | 507 | 1.4% | 55 | 4810 | 1.1% |
| BRAF V600E | 9 | 507 | 1.8% | 107 | 6529 | 1.6% |
| MET exon 14 Splice-site/Deletion | 8 | 507 | 1.6% | 81 | 6529 | 1.2% |
| NTRK1/NTRK2/  NTRK3 Fusion | 1 | 507 | 0.2% | 9 | 4615 | 0.2% |
| ALK Rearrangement | 5 | 507 | 1.0% | 92 | 6463 | 1.4% |
| Any* | 76 | 507 | 15.0% | 1457 | 6529 | 22.3% |

Genomic alteration data were collected for 507 TCGA Lung Adenocarcinoma patients (study id = luad_tcga_pan_can_atlas_2018) and 6529 GENIE Lung Adenocarcinoma patients (GENIE Cohort v5.0-public, cancer type detailed = lung adenocarcinoma). For GENIE patients with multiple samples tested, the first record was chosen to represent this specific patient.

* Patients carrying at least one listed actionable alteration were counted as alteration.
