# Supplementary material for: Precision medicine and actionable alterations in lung cancer: A single institution experience
Source: PLoS One. 2020 Feb 11;15(2):e0228188. doi: 10.1371/journal.pone.0228188 (PMC7012442; doi:10.1371/journal.pone.0228188)
Supplement: S3 Table — (DOCX) [file pone.0228188.s003.docx]

**S3 Table. Multivariate Cox Proportional Hazards Regression Models for Adjusted for Sex, Age, and Smoking Status.**

| **Risk Factor** | **Hazard Ratio (95% CI)** | **P Value** |
| --- | --- | --- |
| Sex, Male vs Female | 1.58 (1.22-2.05) | 0.0005 |
| Age, >=70 vs <70 | 1.29 (0.98-1.70) | 0.070 |
| Smoking Status,  Medium + Heavy vs Never + Light |  |  |
|  | 1.12 (0.83-1.49) | 0.45 |
| EGFR (L858/exon 19 deletion) |  |  |
| Positive vs Tested Negative  EGFR*time | 0.46 (0.30-0.71)  1.02 (1.01-1.03) | 0.0004  0.0011 |
| ALK (Rearrangement) |  |  |
| Positive vs Tested Negative  Not available vs Tested Negative | 0.37 (0.18-0.76)  0.86 (0.54-1.38) | 0.0067  0.53 |
| KRAS |  |  |
| Positive vs Tested Negative  Not available vs Tested Negative | 1.07 (0.75-1.53)  1.15 (0.75-1.78) | 0.72  0.52 |
| TP53 |  |  |
| Positive vs Tested Negative  Not available vs Tested Negative | 1.08 (0.78-1.51)  1.47 (1.01-2.14) | 0.63  0.046 |
