# Supplementary material for: Precision medicine and actionable alterations in lung cancer: A single institution experience
Source: PLoS One. 2020 Feb 11;15(2):e0228188. doi: 10.1371/journal.pone.0228188 (PMC7012442; doi:10.1371/journal.pone.0228188)
Supplement: S4 Table — (DOCX) [file pone.0228188.s004.docx]

**S4 Table.** Cox Proportional Hazards Regression Models for Actionable Genes Adjusted for Sex, Age and Smoking Status.

| **Risk Factor** | **Hazard Ratio (95% CI)** | | **P Value** |
| --- | --- | --- | --- |
| Sex, Male vs Female | | 1.53 (1.19-1.93) | 0.001 |
| Age, >=70 vs <70 | | 1.32 (1.01-1.74) | 0.046 |
| Smoking Status,  Medium + Heavy vs Never + Light | | 1.15 (0.87-1.51) | 0.32 |
|  |  |  |  |
| Actionable Genes, | |  |  |
| Alteration vs Wild-type  Actionable Genes*time | | 0.52 (0.35-0.77)  1.01 (1.00-1.02) | 0.001  0.012 |
